# A specific mutation in *TBL1XR1* causes Pierpont syndrome

**DOI:** 10.1136/jmedgenet-2015-103233

**Published:** 2016-01-14

**Authors:** Charlotte A Heinen, Aldo Jongejan, Peter J Watson, Bert Redeker, Anita Boelen, Olga Boudzovitch-Surovtseva, Francesca Forzano, Roel Hordijk, Richard Kelley, Ann H Olney, Mary Ella Pierpont, G Bradley Schaefer, Fiona Stewart, A S Paul van Trotsenburg, Eric Fliers, John W R Schwabe, Raoul C Hennekam

**Affiliations:** 1 Department of Endocrinology and Metabolism, Academic Medical Centre, University of Amsterdam, Amsterdam, The Netherlands; 2 Department of Paediatric Endocrinology, Emma Children's Hospital, Academic Medical Centre, University of Amsterdam, Amsterdam, The Netherlands; 3 Department of Clinical Epidemiology, Biostatistics and Bioinformatics, Academic Medical Centre, University of Amsterdam, Amsterdam, The Netherlands; 4 Department of Biochemistry, Henry Wellcome Laboratories of Structural Biology, University of Leicester, Leicester, UK; 5 Department of Clinical Genetics, Academic Medical Centre, University of Amsterdam, Amsterdam, The Netherlands; 6 Medical Genetics Unit, Ospedali Galliera, Genova, Italy; 7 Department of Genetics, University of Groningen, University Medical Centre Groningen, Groningen, The Netherlands; 8 Division of Metabolism, Kennedy Krieger Institute, Johns Hopkins University, Baltimore, Maryland, USA; 9 Munroe-Meyer Institute for Genetics and Rehabilitation, University of Nebraska Medical Centre, Omaha, Nebraska, USA; 10 Division of Genetics, Children's Hospitals and Clinics of Minnesota, University of Minnesota, Minneapolis, Minnesota, USA; 11 Division of Medical Genetics, Arkansas Children's Hospital, Little Rock, Arkansas, USA; 12 Division of Medical Genetics, Belfast City Hospital, Belfast, Ireland; 13 Department of Paediatrics, Emma Children's Hospital, Academic Medical Centre, University of Amsterdam, Amsterdam, The Netherlands

**Keywords:** Genetics, Molecular genetics, Psychiatry

## Abstract

**Background:**

The combination of developmental delay, facial characteristics, hearing loss and abnormal fat distribution in the distal limbs is known as Pierpont syndrome. The aim of the present study was to detect and study the cause of Pierpont syndrome.

**Methods:**

We used whole-exome sequencing to analyse four unrelated individuals with Pierpont syndrome, and Sanger sequencing in two other unrelated affected individuals. Expression of mRNA of the wild-type candidate gene was analysed in human postmortem brain specimens, adipose tissue, muscle and liver. Expression of RNA in lymphocytes in patients and controls was additionally analysed. The variant protein was expressed in, and purified from, HEK293 cells to assess its effect on protein folding and function.

**Results:**

We identified a single heterozygous missense variant, c.1337A>C (p.Tyr446Cys), in transducin β-like 1 X-linked receptor 1 (*TBL1XR1*) as disease-causing in all patients. TBL1XR1 mRNA expression was demonstrated in pituitary, hypothalamus, white and brown adipose tissue, muscle and liver. mRNA expression is lower in lymphocytes of two patients compared with the four controls. The mutant TBL1XR1 protein assembled correctly into the nuclear receptor corepressor (NCoR)/ silencing mediator for retinoid and thyroid receptors (SMRT) complex, suggesting a dominant-negative mechanism. This contrasts with loss-of-function germline *TBL1XR1* deletions and other *TBL1XR1* mutations that have been implicated in autism. However, autism is not present in individuals with Pierpont syndrome.

**Conclusions:**

This study identifies a specific *TBL1XR1* mutation as the cause of Pierpont syndrome. Deletions and other mutations in *TBL1XR1* can cause autism. The marked differences between Pierpont patients with the p.Tyr446Cys mutation and individuals with other mutations and whole gene deletions indicate a specific, but as yet unknown, disease mechanism of the TBL1XR1 p.Tyr446Cys mutation.

## Introduction

In 1998 Pierpont and coworkers described two unrelated boys with remarkably similar faces (high forehead, underdeveloped mid-face, narrow palpebral fissures and anteverted nares), short stature, hearing loss, developmental delay and distinctive palmar and plantar fat pads.^1^ Two similar patients were subsequently reported, including one with a choroid plexus papilloma, whereupon the condition was named Pierpont syndrome.^2^ ^3^ While several patients resembling Pierpont syndrome have been reported,^4^ clinical re-evaluation and molecular analyses have shown that they had either Coffin–Siris^5^ or Wiedemann–Steiner syndrome (H. Brunner, personal communication 2013). Initially this caused uncertainty about the phenotype defining Pierpont syndrome, but the characteristics of both Wiedemann–Steiner syndrome and Coffin–Siris syndrome are better known nowadays and allow easy differentiation from the phenotype in the reported patients with Pierpont syndrome.^2^ ^3^ Until now, the cause of Pierpont syndrome has remained unknown. However, de novo autosomal-dominant mutations were suspected to be the most likely cause.^2^

For the present study, we collected DNA from two newly identified and four earlier-reported patients with Pierpont syndrome. We performed whole-exome sequencing in four patients and in the parents of one of them, and identified identical de novo missense mutations in the transducin β-like 1 X-linked receptor 1 (*TBL1XR1*) gene in all four patients. Sanger sequencing of the two newly diagnosed patients revealed the same mutation. TBL1XR1 is part of the nuclear receptor corepressor (NCoR) complex that plays an essential role in gene transcription.

## Methods

### Study cohort

**Figure 1:**
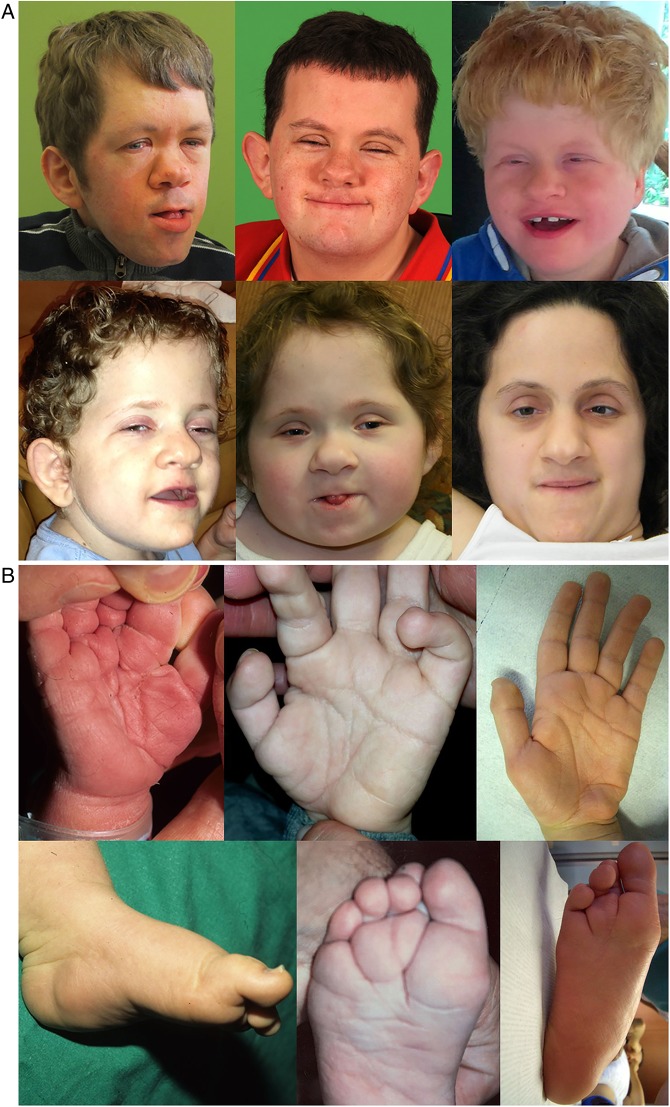
Face and extremity features in individuals with Pierpont syndrome. Note (A) the high forehead, narrow palpebral fissures, flat malae, broad nasal ridge and tip, thin upper vermillion and large ears (upper row, left to right: patients 1, 2 and 3; lower row, left to right: patients 4, 5 and 6); (B) marked grooves and pillowing of hands and feet, and subcalcaneal fat pads (upper row, left to right: patients 2, 4 and 6; lower row, left to right: patients 2, 4 and 6).

The study cohort consisted of six unrelated patients with Pierpont syndrome (table 1). The clinical diagnosis was based on the combination of the facial characteristics, palmar and plantar fat pads, and global developmental delay (figure 1). The description of patients 1–4 has been published.^1–3^

**Table 1:** Main clinical features of presently described individuals with Pierpont syndrome, including updates of the published patients 1 and 2,^1^ 3^2^ and 4^3^

Patient	1	2	3	4	5	6	Total
Age (years)	28	20	12	5.7	10	19	
Gender	M	M	M	M	F	F	4M/2F
Growth parameters at birth*							
Length (cm)	45.7 (<P3)	X	50.0 (P50)	48.5 (P10)	43.0 (<P3)	51 (P75)	
Weight (kg)	3.0 (P25)	3.62 (P50)	2.64 (P10)	2.95 (P25)	2.43 (P3)	2.95 (P25)	
OFC (cm)	35.5 (P75)	X	33.8 (P25)	32.0 (P5)	28.0 (<P3)	33 (P25)	
Growth parameters at age (years)	27	18	12	5.7	10	18	
Height (cm)	147 (<P3)	147 (<P3)	128 (<P3)	100 (<P3)	109 (<P3)	144 (<P3)	
Weight (kg)	36 (<P3)	58 (P10)	30 (P5)	18 (P25)	22 (<P3)	40 (<P3)	
OFC (cm)	53.5 (P10)	44 (<P3)	54 (P50)	45.7 (<P3)	43 (<P3)	56 (P97)	
Intellectual disability†	++	+	++	++	++	+++	6/6
Hypotonia	+	+	+	++	+	+	6/6
Brain imaging	−	−	A	EV, CP	A, EV	A	4/6
High anterior hairline	+	+	+	+	+	+	6/6
Narrow palpebral fissures	+	+	+	−	+	+	5/6
Microcornea‡	−	+	−	−	+	+	3/6
Flat malae	+	+	+	+	+	+	6/6
Broad nasal ridge and tip	+	+	+	+	+	+	6/6
Smooth philtrum/thin vermillion	+	+	+	+	+	+	6/6
Teeth	WS, AE	WS	WS	WS, AE	WS, AE	WS, AS	6/6
Large ears	+	+	+	+	+	+	6/6
Hearing loss§	+	+	+	+	+	−	5/5
Scoliosis	+	++	+	−	++	+	5/6
Short fingers/toes	+	+	+	+	+	+	6/6
Palmar/plantar grooves, pillowing	+	+	+	+	+	+	6/6
Marked foetal finger/toe pads	+	+	+	+	+	+	6/6
Subcalcaneal fat pads	+	+	+	+	+	+	6/6

*Centiles between brackets.

†+IQ 50–60 ++IQ 35–50 +++IQ <35 (IQ data from publications,^1–3^ formal testing in patient 5, and estimated in patient 6).

‡Cornea diameter<10.0 mm.

§Hearing loss was evaluated by audiometry.

+, abnormality present; −, abnormality not present; A, central atrophy; AE, abnormal dental eruption; CP, choroid plexus papilloma; EV, enlarged ventricles; OFC, occipital frontal circumference; WS, widely spaced; X, no data available.

Blood samples were obtained from all patients and their (unaffected) parents. Informed consent for the study was obtained from the parents of all patients. The Medical Ethics Committee of the Academic Medical Centre in Amsterdam (NL45117.018.13) approved the study.

### Molecular analysis

Targeted enrichment and massive parallel sequencing were performed on genomic DNA extracted from circulating leucocytes of four patients and the parents of one of them. Enrichment of the whole exome was performed using the Nimblegen SeqCap EZ Library V.3.0 (Roche). Each captured library was then loaded on a SOLiD5500xl platform (Applied Biosystems) (patient 20120174 and unaffected parents 20112227 and 20112228, and patients 20112226, 20121069 and 20121072). Paired-end and single-end sequence reads were aligned to hg19 using the Lifescope aligner (V.2.5.1) (Applied Biosystems). Presumed PCR duplicates were discarded using Picard Tools (http://picard.sourceforge.net) in case of single-end reads or Lifescope when dealing with paired-end reads. Local realignment and base-quality-score recalibration were performed with the Genome Analysis Toolkit (GATK2 V.2.2-5-g3bf5e3f).^6^

For reads mapping to the human genome reference, mean target region coverage for 20112226 and the trio was 94.5%, with average sequencing depth on target of 92× and for samples 20121069 and 20121072, respectively, 90.5% and 69×. Calls of SNPs and small insertions and deletions (INDELs) were based on 18 unrelated exomes using the GATK Unified Genotyper algorithm, and categorised based on their matching quality, depth of coverage, base quality, the combination of base quality and depth, the position of the alternate allele in the read, and strand bias.^7^ ^8^ Variants were functionally annotated using KGGSeq v0.4 applying available public datasets from the 1000 Genomes Project, NHLBI GO Exome Sequencing Project and dbSNP (V.137), and predictions were made regarding probabilities of being disease causing. Only variants passing all applied GATK filters, predicted to be a de novo mutation within the trio and disease-causing by KGGSeq were retained.

### Protein characterisation

The structure of the WD40 domain of TBL1XR1 was obtained from the Protein Data Bank (PDB accession code 4LG9). For expression in mammalian cells, constructs of TBL1XR1, HDAC3 and GPS2-SMRT chimaera were cloned into the pcDNA3 vector. Transient transfections and protein purifications were performed as described elsewhere.^9^ For the TBL1XR1/HDAC3/GPS2-SMRT chimaera complex, the GPS2-SMRT chimaera contained an N-terminal 10×His-3×Flag tag and a tobacco etch virus (TEV) protease cleavage site. HEK293F cells (Invitrogen) were cotransfected with mixtures of both tagged and untagged constructs using polyethylenimine (PEI) (Sigma). To transfect 60 mL of cells, 60 µg DNA total was diluted in 6 mL of phosphate-buffered saline (PBS) (Sigma) and vortexed briefly; 240 µL of 0.5 mg/mL PEI was added, then vortexed briefly, and incubated for 20 min at room temperature, then added to 60 mL cells (final density 1×10^6^ cells/mL). Cells were harvested 48 h after transfection. For the interaction studies the cells were lysed by sonication in 50 mM Tris/Cl pH 7.5, 100 mM potassium acetate, 5% v/v glycerol, 0.3% v/v Triton X-100 and Roche complete protease inhibitor (buffer A); insoluble material was removed by centrifugation. The complex was bound to Flag resin (Sigma), washed three times with buffer A, three times with buffer B (50 mM Tris/Cl pH 7.5, 300 mM potassium acetate, 5% v/v glycerol) and three times with buffer C (50 mM Tris/Cl pH 7.5, 50 mM potassium acetate, 5% v/v glycerol, 0.5 mM tris (carboxyethyl) phosphine (TCEP)). The complex was eluted from the resin by overnight cleavage at 4°C with TEV protease in buffer C.

### mRNA expression

We analysed TBL1XR1 mRNA expression in brain tissue (hypothalamus; pituitary gland), in white and brown adipose tissue, in muscle tissue and in liver tissue. In addition we analysed *TBL1XR1* RNA expression in lymphocytes of two patients (aged 13 and 20 years, respectively) and four controls (age between 25 and 30 years).

#### Tissues

Three hypothalami and pituitaries were obtained from the Netherlands Brain Bank in accordance with permission for brain autopsy and the use of human brain material and clinical information for research purposes. Three unfixed, frozen (−80°C) hypothalamus–pituitary specimens were used for mRNA expression. The paraventricular nucleus (PVN) region was cut in serial, coronal 50 μm sections from unfixed frozen hypothalami on a cryostat and the PVN area macroscopically dissected, collected, and stored at −80°C until processing, as previously reported.^10^ RNA was extracted from the PVN and from homogenised pituitaries using TriReagent (Sigma) per manufacturer's instructions, followed by DNase treatment (Qiagen GmbH, Germany). cDNA was synthesised with an Applied Biosystem Kit. White adipose tissue cDNA was kindly provided by Drs M Serlie and M Kilicarslan (Department of Endocrinology, AMC, Amsterdam) and synthesised from RNA isolated from subcutaneous, periumbilical adipose tissue biopsies from healthy lean men under local anaesthesia (approved by the Medical Ethics Committee of the Academic Medical Centre in Amsterdam). Liver tissue cDNA was kindly provided by Drs M Serlie and P Gilijamse (Department of Endocrinology, AMC, Amsterdam) and synthesised from RNA isolated from liver tissue biopsies obtained during gastric bypass surgery (approved by the Medical Ethics Committee of the Academic Medical Centre in Amsterdam). RNA was isolated using TRIzol reagent (Invitrogen, Breda, the Netherlands) followed by the NucleoSpin RNA extraction kit (Machterey & Nagel GmbH, Duren) and DNase treatment (Ambion, Carlsbad, California, USA). cDNA was synthesised using Transcriptor First Strand cDNA Synthesis Kit (Roche) according to the manufacturer's instructions. Brown adipose tissue biopsies were taken during thyroid surgery (approved by the Medical Ethics Committee of the University Medical Centre Maastricht) and the samples were kindly provided by Drs E Nascimento, E Broeders, N Bouvy, P Schrauwen and W van Marken Lichtenbelt (Maastricht University). RNA was isolated on the Magna Pure (Roche Molecular Biochemicals, Mannheim, Germany) using the Magna Pure LC mRNA tissue kit. The protocol and buffers supplied with the corresponding kit were applied. cDNA synthesis was performed using the Transcriptor cDNA Synthesis Kit for RT-PCR with oligo d(T) primers (Roche Molecular Biochemicals, Mannheim, Germany). Muscle cDNA was commercially available and obtained from Clontech, Takara (Mountain View, California, USA). RNA from whole blood of four healthy controls and two patients was isolated using the High Pure RNA Isolation Kit (Roche Molecular Biochemicals, Mannheim, Germany) according to the manufacturer's protocol. cDNA synthesis was performed using the Transcriptor cDNA Synthesis Kit for RT-PCR with oligo d(T) primers (Roche Molecular Biochemicals, Mannheim, Germany). From every sample a –RT reaction was performed in order to check for genomic DNA contamination.

#### PCR

Primers were designed to amplify TBL1XR1 transcript (NM_024665.4, F: 5′-CCATGGCCAGTCCACTACAG-3, R: 5′-TCCAGCACTTGGTGAACAGA-3′), product size 126 bp, and annealing temperature 65°C*.* Real-time PCR was performed using the Lightcycler480 and Lightcycler480SybrGreen I Master mix (Roche Molecular Biochemicals, Mannheim). Melting curve analysis was performed and product size was determined by DNA gel analysis. All samples contain mRNA as checked by HPRT expression (hypoxanthine phosphoribosyl transferase, a housekeeping gene).^10^ Expression levels in whole blood were quantified using the LinReg software.^11^ The mean efficiency was calculated for each assay and samples that had a deviation of more than 5% were excluded. Calculated values were normalised by HPRT expression.

## Results

### Study cohort

Patient 5 was the term, first-born child of healthy, non-consanguineous parents. She had intrauterine growth retardation, was hypotonic at birth, and had bilateral hip dislocations. She experienced feeding difficulties with gastrostomy placement in infancy, and followed a markedly delayed motor and cognitive development. She was able to walk with assistance, and had no speech. Formal cognitive testing at age 7 years showed her IQ to be 45. Hearing loss was detected in the first year of life. She gradually developed a progressive thoracolumbar scoliosis requiring rod placement at age 10 years. Her postnatal growth in height and of skull circumference was decreased. Onset of menses was at the age of 11 years. Both her unusual face and the abnormal creases of palms and soles were evident at birth. She has always had mild fat pads anteromedial to both heels.

Patient 6 was the first-born child of healthy, non-consanguineous parents. She was remarkably hypotonic at birth. At that time it was noticed that she had unusual facial morphology, broad thumbs, deep pillowing of palms and soles, and bilateral talipes. Her development was markedly delayed from early on: she never developed any speech and had no sphincter control. Formal cognitive testing was not possible, but her IQ was estimated to be below 35 at age 18 years. She had increasingly decreased growth in height, a relatively large head, low body weight and little subcutaneous fat tissue. She gradually developed pectus excavatum and thoracic scoliosis, but otherwise had no significant health problems.

### Molecular analysis

**Figure 2:**
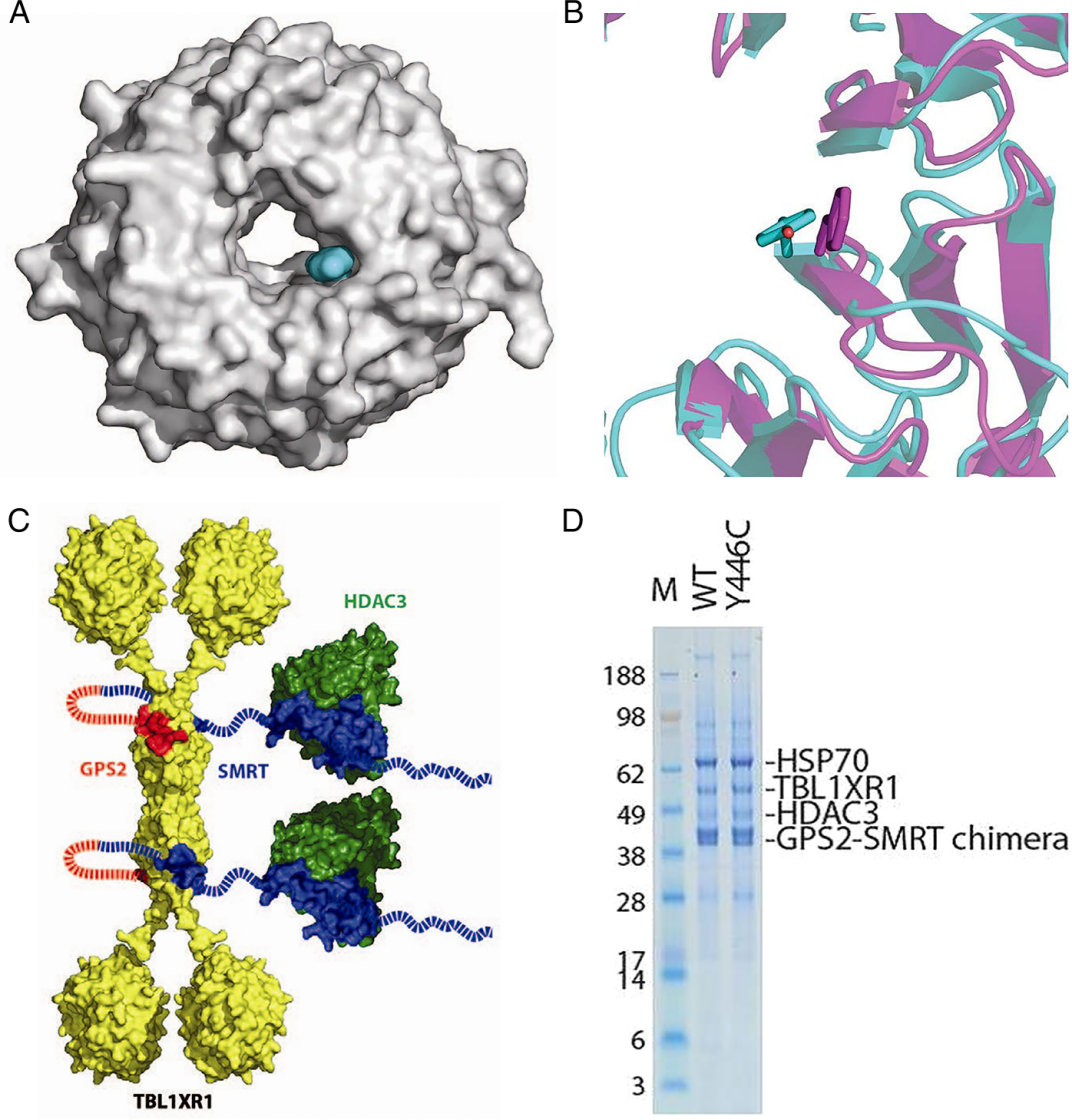
(A) Surface representation of the transducin β-like 1 X-linked receptor 1 (TBL1XR1) WD40 domain (PDB ID 4LG9), the mutated residue (Y446) is shown in cyan. (B) Comparison of Y446 in human TBL1XR1 (cyan) and f446 in yeast (purple). (C) Representation of the TBL1XR1/HDAC3/GPS2-SMRT chimaera complex. (D) sodium dodecyl sulfate polyacrylamide gel electrophoresis (SDS–PAGE) of the purification of the wild-type and mutant TBL1XR1/HDAC3/GPS2-SMRT chimaera complex.

Whole-exome sequencing yielded a single missense mutation c.1337A>C in *TBL1XR1* located at 3q26.32, resulting in the amino acid substitution p.Tyr446Cys (Y446C) in all four patients studied, but not in the parents of one of them (see online supplementary table S1). No other potentially pathogenic variant in the same gene was present in all four patients. Sanger sequencing demonstrated the same mutation in the two other patients. All other parents tested negative for the mutation using Sanger sequencing, indicating de novo occurrence. The mutation was at an evolutionary conserved position (figure 2A) and absent in control populations (dbSNP, 1000 genomes, NHBLI, ESP, GoNL).

### Stability of mutated complexes

TBL1XR1 is a highly conserved protein found in all eukaryotes (see online supplementary figure S1). It contains two structured domains: an amino-terminal domain that mediates tetramerisation of the protein and a carboxy-terminal WD40 domain. The TBL1XR1 tyrosine-to-cysteine mutation identified here is located in the WD40 domain on one side of the inner surface of the WD40 ring (figure 2A). Y446 is largely exposed to solvent, and mutation to cysteine would not be expected to significantly perturb the structure of the domain. A tyrosine in this position is found in nearly all TBL1XR1 proteins, although the equivalent residue is a phenylalanine in the homologous Sif2 protein from *Saccharomyces cerevisiae*. The structure of the WD40 domain from Sif2 has also been reported.^12^ Despite relatively low sequence identity between TBL1XR1 and Sif2, the structures of their WD40 rings itself are similar. Furthermore, the yeast residue equivalent to Y446, f446, adopts a very similar conformation (figure 2B) suggesting a conserved function for this largely non-polar amino acid. To confirm that the Y446C mutation does not grossly perturb the fold and behaviour of TBL1XR1 we examined the ability of the TBL1XR1 to assemble correctly with the GPS2:SMRT:HDAC3 complex. As predicted, the mutant protein was readily expressed and purified and assembled correctly into the TBL1XR1:GPS2:SMRT:HDAC3 complex (figure 2C and D), suggesting that the molecular pathology of the Y446C mutation is an impaired protein–protein interaction with an as yet unidentified molecular partner rather than a failure to fold correctly.

### mRNA expression

**Figure 3:**
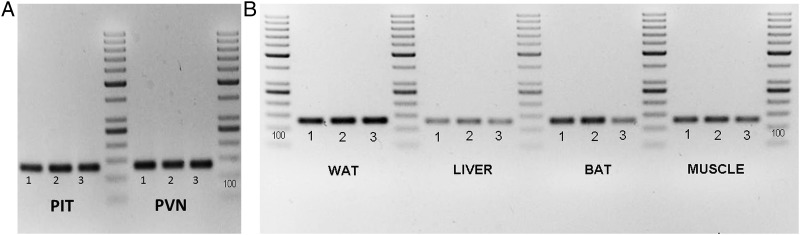
(A) Transducin β-like 1 X-linked receptor 1 (TBL1XR1) mRNA expression in human pituitary and hypothalamic PVN. (B) TBL1XR1 mRNA expression in human white and brown adipose tissue, liver and muscle tissue. TBL1XR1 transcript PCR product on 2% agarose gel. The expected product is 126 bp. BAT, brown adipose tissue; PIT, pituitary; PVN, paraventricular nucleus; WAT, white adipose tissue.

**Figure 4:**
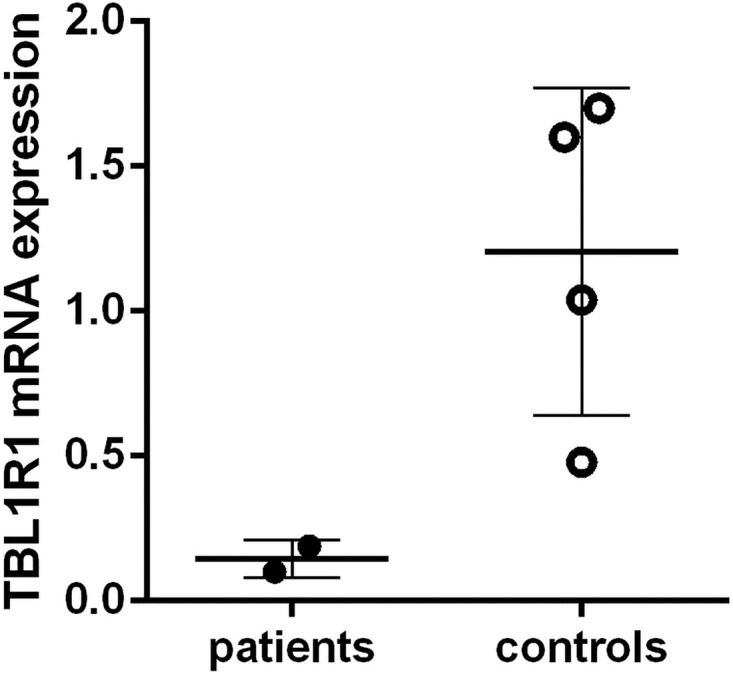
Relative expression of transducin β-like 1 X-linked receptor 1 (*TBL1XR1*) mRNA to hypoxanthine phosphoribosyl transferase (used as reference gene) in leucocytes of patients (closed circles) and controls (open circles). Individual values are depicted and mean values ±SD is represented by a solid line.

TBL1XR1 mRNA expression was well visible in the pituitary and the PVN area of the hypothalamus, as well as in liver and muscle tissue and both white and brown adipose tissue, fitting the clinical symptomatology of Pierpont syndrome (figure 3A, B). TBL1XR1 mRNA expression in whole blood was lower in patients compared with controls (figure 4). The small number of patients available for analysis precludes statistical analysis to determine whether this difference is significant.

## Discussion

In this study, we found a single *TBL1XR1* missense mutation in six patients with Pierpont syndrome that was absent in their unaffected parents.

**Figure 5:**
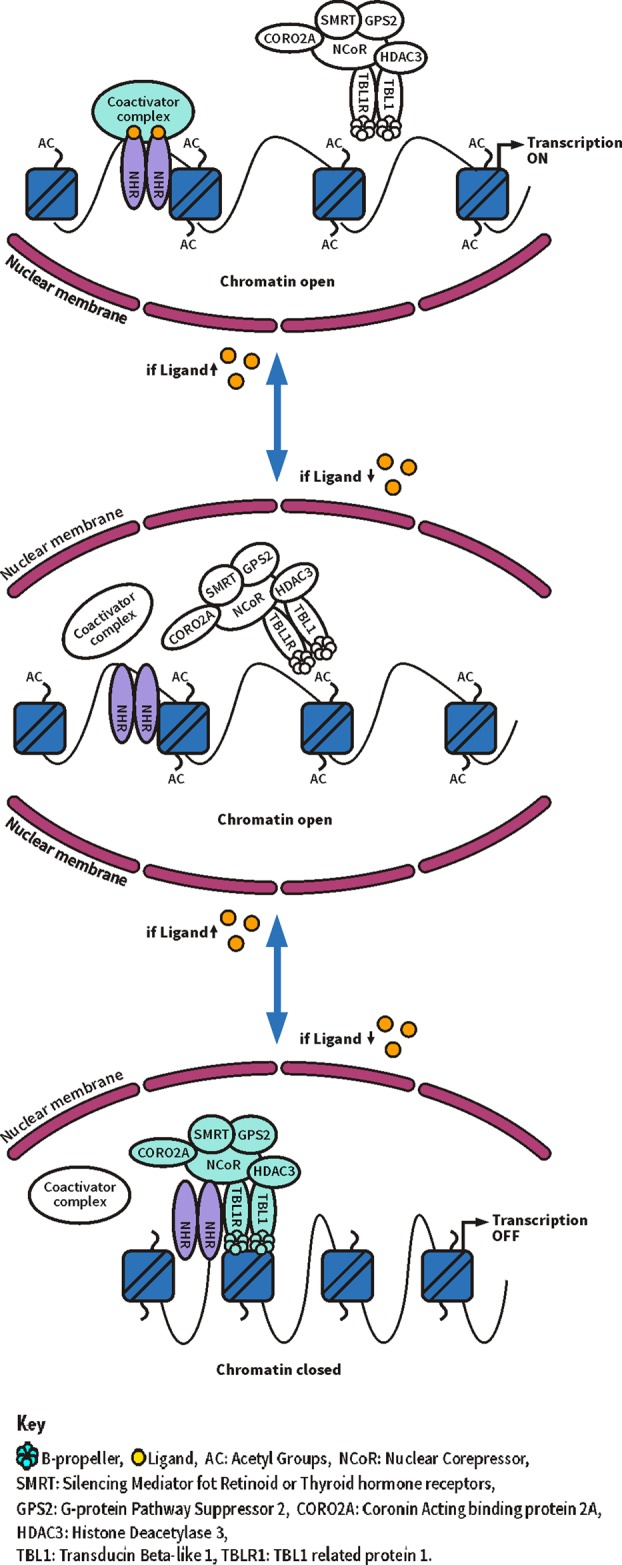
Schematic model of transcriptional regulation by the SMRT/nuclear receptor corepressor (NCoR) complex.

TBL1XR1, a member of the WD40 repeat-containing protein family, is composed of 18 exons.^13^ The product of *TBL1XR1*, TBL1XR1 (or TBLR1; 55 595 Da; 514 amino acids), contains a carboxy-terminal WD40 domain containing eight WD40 repeats and an amino-terminal LisH domain that mediates tetramerisation of the protein and its interactions with NCoR/SMRT and GPS2.^13^ ^14^ TBL1XR1 is an essential component of the NCoR/SMRT corepressor complex (figure 2C), which interacts with nuclear hormone receptors, a family of ligand-dependent transcription factors involved in regulation of gene transcription (figure 5).^15^ When bound to unliganded nuclear hormone receptors, corepressors mediate silencing of gene transcription by recruiting chromatin-modifying enzymes. When a nuclear hormone receptor is liganded, corepressors dissociate to relieve repression of transcription. In negatively regulated target genes, corepressors are essential for activation of transcription.^16^ The WD40 repeats in TBL1XR1 are thought to be involved in the interaction of the corepressor complex with histones stabilising the association of the complex with the chromatin.^13^ ^17^ TBL1XR1 also may play a regulatory role in the NF-κB pathway and *Wnt*-mediated transcription. NF-kB transcription requires IKKα to phosphorylate SMRT on chromatin, which recruits TBL1XR1 to the gene promoter. During depletion of TBL1XR1, NF-kB transcription and cell survival are compromised.^17^ ^18^ TBL1XR1 also recruits β-catenin to the Wnt target-gene promoter. In the presence of TBL1XR1, β-catenin is able to remove corepressors from the promoter of Wnt target genes by competitive binding, thereby activating transcription.^19^

*TBL1XR1* mutations have been implicated in several phenotypes. Recurrent *TBL1XR1* mutations have been described in DNA of lymphatic malignancies, including primary central nervous system lymphomas,^20^ ^21^ acute lymphoblastic leukaemia^22^ and Sézary syndrome.^23^ The precise mechanisms by which *TBL1XR1* mutations contribute to tumourigenesis remain unclear, but it has been hypothesised that loss of *TBL1XR1* could compromise the ability of corepressor complexes to inhibit receptor activity, leading to increased activation of receptor target genes.^22^ In addition, it has been suggested that *TBL1XR1* might act as a tumour-suppressor gene in the lymphatic system,^21^ and upregulates VEGF-C inducing lymphangiogenesis in oesophageal squamous cell carcinoma.^24^ Contrarily, *TBL1XR1* has been nominated as a novel breast cancer oncogene,^25^ indicating that any role of *TBL1XR1* in tumourigenesis is tissue-specific.

Whole-exome sequencing of 209 children with autism spectrum disorder (ASD) and their parents showed a de novo p.Leu282Pro mutation in TBL1XR1 in one child, who also had intellectual disability.^26^ Subsequently, evaluation of 44 candidate genes in 2494 ASD cases identified two de novo TBL1XR1 mutations (p.Leu282Pro, p.Ile397SerfsX19).^27^ Apart from intellectual disability, however, these patients had no features in common with Pierpont syndrome (B O'Roak and E Eichler, personal communication 2013). Similarly, a non-dysmorphic patient with intellectual disability, autism and West syndrome was found to have a de novo p.Gly70Asp mutation in *TBL1XR1.*^28^ Three patients, including a mother and child, have been described with a deletion involving only *TBL1XR1* in patients who had mild to moderate intellectual disability without autistic behaviour or manifestations of Pierpont syndrome.^29^ ^30^ In addition, three cases with small deletions involving *TBL1XR1* and other genes are included in the Decipher database (http://decipher.sanger.ac.uk/). All had intellectual disability, ASD or ASD-like behaviour but lacked physical signs of Pierpont syndrome (Z Stark, M Decamp, B Dallapiccola, personal communications, 2014). In one additional published patient the phenotype was attributed to a 2.2 Mb deletion at 3q26.3 involving *TBL1XR1*, but an updated annotation showed that the deletion did not encompass *TBL1XR1.*^31^ This suggests that the phenotype of individuals with a microdeletion 3q26.3 is caused by a loss of function of *TBL1XR1*, and consists of intellectual disability and frequently ASD, and a phenotype that shows no resemblance to Pierpont syndrome. It remains at present uncertain whether the abundant hypothalamic and pituitary mRNA expression of wild-type TBL1XR1 reported here is related to the intellectual disability in individuals with deletions.

The differences in phenotype in subjects with the Y446C mutation compared with subjects with whole-gene deletions or other *TBL1XR1* mutations suggest different pathogenic mechanisms. The Y446C mutation could act in a dominant negative way, in agreement with our finding that the mutant TBL1XR1 Y446C protein is able to assemble into the HDAC3 corepressor complex. Since TBL1XR1 Y446C can form hetero-tetramers with wild-type TBL1XR1, TBL1XR1 Y446C will likely be present in most HDAC3 corepressor complexes, and the impaired or inappropriate protein–protein interactions with an as yet unidentified molecular partner will likely be present ubiquitously.

In conclusion, one specific *TBL1XR1* missense mutation is responsible for the phenotype in individuals with Pierpont syndrome. The difference in phenotype between non-ASD Pierpont patients with the Y446C mutation in *TBL1XR1* and individuals with a complete deletion or other mutation of *TBL1XR1* suggests mutation-specific mechanisms of pathogenesis for ASD. Further studies of the pathogenesis in individuals with deletions and various mutations in *TBL1XR1* could yield useful insights into the pathogenesis of ASD.

## Supplementary Material

Web supplement

## References

[1] Pierpont M, Stewart F, Gorlin R Plantar lipomatosis, unusual facial phenotype and developmental delay: a new MCA/MR syndrome. Am J Med Genet 1998;75:18–21. 10.1002/(SICI)1096-8628(19980106)75:1<18::AID-AJMG5>3.0.CO;2-M 9450851

[2] Oudesluijs GG, Hordijk R, Boon M, Sijens PE, Hennekam RC Plantar lipomatosis, unusual facies, and developmental delay: confirmation of Pierpont syndrome. Am J Med Genet 2005;137A:77–80. 10.1002/ajmg.a.30863 16007632

[3] Vadivelu S, Edelman M, Schneider SJ, Mittler MA Choroid plexus papilloma and Pierpont syndrome. J Neurosurg Pediatr 2013;11:115–8. 10.3171/2012.10.PEDS12219 23176139

[4] Wright EM, Suri M, White SM, de Leeuw N, Vulto-van Silfhout AT, Stewart F, McKee S, Mansour S, Connell FC, Chopra M, Kirk EP, Devriendt K, Reardon W, Brunner H, Donnai D Pierpont syndrome: a collaborative study. Am J Med Genet 2011;155A:2203–11. 10.1002/ajmg.a.34147 21834056 PMC4495254

[5] Sim JC, White SM, Fitzpatrick E, Wilson GR, Gillies G, Pope K, Mountford HS, Torring PM, McKee S, Vulto-van Silfhout AT, Jhangiani SN, Muzny DM, Leventer RJ, Delatycki MB, Amor DJ, Lockhart PJ Expanding the phenotypic spectrum of ARID1B-mediated disorders and identification of altered cell-cycle dynamics due to ARID1B haploinsufficiency. Orphanet J Rare Dis 2014;9:43 10.1186/1750-1172-9-43 24674232 PMC4022252

[6] McKenna A, Hanna M, Banks E, Sivachenko A, Cibulskis K, Kernytsky A, Garimella K, Altshuler D, Gabriel S, Daly M, DePristo MA The Genome Analysis Toolkit: a MapReduce framework for analyzing next-generation DNA sequencing data. Genome Res 2010;20:1297–303. 10.1101/gr.107524.110 20644199 PMC2928508

[7] DePristo MA, Banks E, Poplin R, Garimella KV, Maguire JR, Hartl C, Philippakis AA, del Angel G, Rivas MA, Hanna M, McKenna A, Fennell TJ, Kernytsky AM, Sivachenko AY, Cibulskis K, Gabriel SB, Altshuler D, Daly MJ A framework for variation discovery and genotyping using next-generation DNA sequencing data. Nat Genet 2011;43:491–8. 10.1038/ng.806 21478889 PMC3083463

[8] Li MX, Gui HS, Kwan JS, Bao SY, Sham PC A comprehensive framework for prioritizing variants in exome sequencing studies of Mendelian diseases. Nucleic Acids Res 2012;40:e53 10.1093/nar/gkr1257 22241780 PMC3326332

[9] Watson PJ, Fairall L, Santos GM, Schwabe JW Structure of HDAC3 bound to co-repressor and inositol tetraphosphate. Nature 2012;481:335–40. 10.1038/nature10728 22230954 PMC3272448

[10] Bisschop PH, Dekker MJ, Osterthun W, Kwakkel J, Anink JJ, Boelen A, Unmehopa UA, Koper JW, Lamberts SW, Stewart PM, Swaab DF, Fliers E Expression of 11beta-hydroxysteroid dehydrogenase type 1 in the human hypothalamus. J Neuroendocrinol 2013;25:425–32. 10.1111/jne.12017 23286317

[11] Ramakers C, Ruijter JM, Deprez RH, Moorman AF Assumption-free analysis of quantitative real-time polymerase chain reaction (PCR) data. Neurosci Lett 2003;339:62–6. 10.1016/S0304-3940(02)01423-4 12618301

[12] Cerna D, Wilson DK The structure of Sif2p, a WD repeat protein functioning in the SET3 corepressor complex. J Mol Biol 2005;351:923–35. 10.1016/j.jmb.2005.06.025 16051270

[13] Zhang XM, Chang Q, Zeng L, Gu J, Brown S, Basch RS TBLR1 regulates the expression of nuclear hormone receptor co-repressors. BMC Cell Biol 2006;7:31 10.1186/1471-2121-7-31 16893456 PMC1555579

[14] Oberoi J, Fairall L, Watson PJ, Yang JC, Czimmerer Z, Kampmann T, Goult BT, Greenwood JA, Gooch JT, Kallenberger BC, Nagy L, Neuhaus D, Schwabe JW Structural basis for the assembly of the SMRT/NCoR core transcriptional repression machinery. Nat Struct Mol Biol 2011;18:177–84. 10.1038/nsmb.1983 21240272 PMC3232451

[15] Hu X, Lazar MA Transcriptional repression by nuclear hormone receptors. Trends Endocrinol Metab 2000;11:6–10. 10.1016/S1043-2760(99)00215-5 10652499

[16] Tagami T, Madison LD, Nagaya T, Jameson JL Nuclear receptor corepressors activate rather than suppress basal transcription of genes that are negatively regulated by thyroid hormone. Mol Cell Biol 1997;17:2642–8. 10.1128/MCB.17.5.2642 9111334 PMC232114

[17] Perissi V, Aggarwal A, Glass CK, Rose DW, Rosenfeld MG A corepressor/coactivator exchange complex required for transcriptional activation by nuclear receptors and other regulated transcription factors. Cell 2004;116:511–26. 10.1016/S0092-8674(04)00133-3 14980219

[18] Hoberg JE, Yeung F, Mayo MW SMRT derepression by the IkappaB kinase alpha: a prerequisite to NF-kappaB transcription and survival. Mol Cell 2004;16:245–55. 10.1016/j.molcel.2004.10.010 15494311

[19] Li J, Wang CY TBL1-TBLR1 and beta-catenin recruit each other to Wnt target-gene promoter for transcription activation and oncogenesis. Nat Cell Biol 2008;10:160–9. 10.1038/ncb1684 18193033

[20] Braggio E, McPhail ER, Macon W, Lopes MB, Schiff D, Law M, Fink S, Sprau D, Giannini C, Dogan A, Fonseca R, O'Neill BP Primary central nervous system lymphomas: a validation study of array-based comparative genomic hybridization in formalin-fixed paraffin-embedded tumor specimens. Clin Cancer Res 2011;17:4245–53. 10.1158/1078-0432.CCR-11-0395 21562036 PMC3131452

[21] Gonzalez-Aguilar A, Idbaih A, Boisselier B, Habbita N, Rossetto M, Laurenge A, Bruno A, Jouvet A, Polivka M, Adam C, Figarella-Branger D, Miquel C, Vital A, Ghesquières H, Gressin R, Delwail V, Taillandier L, Chinot O, Soubeyran P, Gyan E, Choquet S, Houillier C, Soussain C, Tanguy ML, Marie Y, Mokhtari K, Hoang-Xuan K Recurrent mutations of MYD88 and TBL1XR1 in primary central nervous system lymphomas. Clin Cancer Res 2012;18:5203–11. 10.1158/1078-0432.CCR-12-0845 22837180

[22] Parker H, An Q, Barber K, Case M, Davies T, Konn Z, Stewart A, Wright S, Griffiths M, Ross FM, Moorman AV, Hall AG, Irving JA, Harrison CJ, Strefford JC The complex genomic profile of ETV6-RUNX1 positive acute lymphoblastic leukemia highlights a recurrent deletion of TBL1XR1. Genes Chromosomes Cancer 2008;47:1118–25. 10.1002/gcc.20613 18767146

[23] Andersson E, Eldfors S, Edgren H, Ellonen P, Väkevä L, Ranki A, Mustjoki S Novel TBL1XR1, EPHA7 and SLFN12 mutations in a Sezary syndrome patient discovered by whole exome sequencing. Exp Dermatol 2014;23:366–8. 10.1111/exd.12405 24689486

[24] Liu L, Lin C, Liang W, Wu S, Liu A, Wu J, Zhang X, Ren P, Li M, Song L TBL1XR1 promotes lymphangiogenesis and lymphatic metastasis in esophageal squamous cell carcinoma. Gut 2015;64:26–36. 10.1136/gutjnl-2013-306388 24667177

[25] Kadota M, Sato M, Duncan B, Ooshima A, Yang HH, Diaz-Meyer N, Gere S, Kageyama S, Fukuoka J, Nagata T, Tsukada K, Dunn BK, Wakefield LM, Lee MP Identification of novel gene amplifications in breast cancer and coexistence of gene amplification with an activating mutation of PIK3CA. Cancer Res 2009;69:7357–65. 10.1158/0008-5472.CAN-09-0064 19706770 PMC2745517

[26] O'Roak BJ, Vives L, Girirajan S, Karakoc E, Krumm N, Coe BP, Levy R, Ko A, Lee C, Smith JD, Turner EH, Stanaway IB, Vernot B, Malig M, Baker C, Reilly B, Akey JM, Borenstein E, Rieder MJ, Nickerson DA, Bernier R, Shendure J, Eichler EE Sporadic autism exomes reveal a highly interconnected protein network of de novo mutations. Nature 2012;485:246–50. 10.1038/nature10989 22495309 PMC3350576

[27] O'Roak BJ, Vives L, Fu W, Egertson JD, Stanaway IB, Phelps IG, Carvill G, Kumar A, Lee C, Ankenman K, Munson J, Hiatt JB, Turner EH, Levy R, O'Day DR, Krumm N, Coe BP, Martin BK, Borenstein E, Nickerson DA, Mefford HC, Doherty D, Akey JM, Bernier R, Eichler EE, Shendure J Multiplex targeted sequencing identifies recurrently mutated genes in autism spectrum disorders. Science 2012;338:1619–22. 10.1126/science.1227764 23160955 PMC3528801

[28] Saitsu H, Tohyama J, Walsh T, Kato M, Kobayashi Y, Lee M, Tsurusaki Y, Miyake N, Goto Y, Nishino I, Ohtake A, King MC, Matsumoto N A girl with West syndrome and autistic features harboring a de novo TBL1XR1 mutation. J Hum Genet 2014;59:581–3. 10.1038/jhg.2014.71 25102098

[29] Tabet AC, Leroy C, Dupont C, Serrano E, Hernandez K, Gallard J, Pouvreau N, Gadisseux JF, Benzacken B, Verloes A De novo deletion of TBL1XR1 in a child with non-specific developmental delay supports its implication in intellectual disability. Am J Med Genet 2014;164A:2335–7. 10.1002/ajmg.a.36619 24891185

[30] Pons L, Cordier MP, Labalme A, Till M, Louvrier C, Schluth-Bolard C, Lesca G, Edery P, Sanlaville D A new syndrome of intellectual disability with dysmorphism due to TBL1XR1 deletion. Am J Med Genet A 2015;167A:164–8. 10.1002/ajmg.a.36759 25425123

[31] Millson A, Lagrave D, Willis MJ, Rowe LR, Lyon E, South ST Chromosomal loss of 3q26.3–3q26.32, involving a partial neuroligin 1 deletion, identified by genomic microarray in a child with microcephaly, seizure disorder, and severe intellectual disability. Am J Med Genet 2012;158A:159–65. 10.1002/ajmg.a.34349 22106001

