# Supplementary material for: A specific mutation in TBL1XR1 causes Pierpont syndrome
Source: J Med Genet. 2016 Jan 14;53(5):330–7. doi: 10.1136/jmedgenet-2015-103233 (PMC4853543; doi:10.1136/jmedgenet-2015-103233)
Supplement: Web supplement [file jmedgenet-2015-103233-s1.pdf]

SUPPLEMENTARY INFORMATION

Charlotte A. Heinen[1,2], Aldo Jongejan[3], Peter J. Watson[4], Bert Redeker[5], Anita Boelen[1], Olga Boudzovitch-Surovtseva[1], Francesca Forzano[6], Roel Hordijk[7], Richard Kelley[8], Ann H. Olney,[9] Mary Ella Pierpont[10], G. Bradley Schaefer[11], Fiona Stewart[12], A.S. Paul van Trotsenburg[2], Eric Fliers[1], John W.R. Schwabe[4], Raoul C. Hennekam[13]

1.Deparment of Endocrinology and Metabolism, Academic Medical Centre, University of Amsterdam, Amsterdam, The Netherlands
2. Department of Paediatric Endocrinology, Emma Children's Hospital, Academic Medical Centre, University of Amsterdam, Amsterdam, The Netherlands
3. Department of Clinical epidemiology, Biostatistics and Bioinformatics, Academic Medical Centre, University of Amsterdam, Amsterdam, the Netherlands
4. Henry Wellcome Laboratories of Structural Biology, Department of Biochemistry, University of Leicester, Leicester, U.K.
5. Department of Clinical Genetics, Academic Medical Centre, University of Amsterdam, Amsterdam, The Netherlands
6. Medical Genetics Unit, Ospedali Galliera, Genova, Italy
7. Department of Genetics, University of Groningen, University Medical Centre Groningen, Groningen, the Netherlands
8. Division of Metabolism, Kennedy Krieger Institute, Johns Hopkins University, Baltimore, MD, USA
9. Munroe-Meyer Institute for Genetics and Rehabilitation, University of Nebraska Medical Centre, Omaha, NE, USA
10. Division of Genetics, Children's Hospitals and Clinics of Minnesota, University of Minnesota, Minneapolis, MN, USA
11. Division of Medical Genetics, Arkansas Children's Hospital, Little Rock, AR, USA
12. Division of Medical Genetics, Belfast City Hospital, Belfast, Ireland
13. Department of Paediatrics, Emma Children's Hospital, Academic Medical Centre, University of Amsterdam, Amsterdam, The Netherlands

On-line Methods

Exome sequencing and sequence data analysis. Targeted enrichment and massively parallel sequencing were performed on genomic DNA extracted from circulating leukocytes. Enrichment of the whole exome was performed using the Nimblegen SeqCap EZ Library v3.0 (Roche). Each captured library was then loaded on a SOLiD5500xl (Applied Biosystems) (20120174 and unaffected parents (20112227 and 20112228), 20112226, 20121069 and 20121072) platforms. Paired-end and single-end sequence reads were aligned to hg19 using the Lifescope aligner (v2.5.1) (Applied Biosystems). Presumed PCR duplicates were discarded using Picard Tools (http://picard.sourceforge.net) in case of single-end reads or Lifescope when dealing with paired-end reads. Local realignment and base-quality-score recalibration were performed with the Genome Analysis Toolkit (GATK v2.2-5-g3bf5e3f).[1]

Following reads mapping to the human genome reference, mean target region coverage for 20112226 and the trio was 94.5%, with average sequencing depth on target of 92x and for samples 20121069 and 20121072 resp. 90.5% and 69x. SNPs and small INDELs were called in the presence of 18 unrelated exomes using the GATK Unified Genotyper algorithm[2], and categorized based on their matching quality (MQ0 >= 4 && ((MQ0 / (1.0 * DP)) > 0.1) -> "HARD_TO_VALIDATE"), depth of coverage (DP < 10 : “VeryLowCoverage", DP > 10 && DP < 16 " : "LowCoverage"), base quality (QUAL < 30.0 : "VeryLowQual", QUAL > 30.0 && QUAL < 50.0 : "LowQual"), the combination of base quality and depth (QD < 2.0 :

"QDFilter"), the position of the alternate allele in the read (ReadPosRankSum < -20.0 : "ReadPosFilter") and strand bias (FS > 200.0 : "FSFilter"). Variants were functionally annotated using KGGSeq v0.4 applying available public datasets from the 1000 Genomes Project, NHLBI GO Exome Sequencing Project and dbSNP (v137) and predictions were made regarding probabilities of being disease causing. Only variants passing all the applied GATK filters, predicted to be a *de novo* mutation within the trio and disease-causing by KGGSeq were retained[3].

References

1. McKenna A, Hanna M, Banks E, Sivachenko A, Cibulskis K, Kernytsky A, Garimella K, Altshuler D, Gabriel S, Daly M, DePristo MA. The Genome Analysis Toolkit: a MapReduce framework for analyzing next-generation DNA sequencing data. Genome Res 2010;20:1297-303.
2. DePristo MA, Banks E, Poplin R, Garimella KV, Maguire JR, Hartl C, Philippakis AA, del Angel G, Rivas MA, Hanna M, McKenna A, Fennell TJ, Kernytsky AM, Sivachenko AY, Cibulskis K, Gabriel SB, Altshuler D, Daly MJ. A framework for variation discovery and genotyping using next-generation DNA sequencing data. Nat Genet 2011;43:491-8.
3. Li MX, Gui HS, Kwan JS, Bao SY, Sham PC. A comprehensive framework for prioritizing variants in exome sequencing studies of Mendelian diseases. Nucleic Acid Res 2012;40:e53.

Supplementary Table S1. Whole exome sequencing data output.

	20112226	0112227	0112228	20120174	20121069	20121072
Target region coverage[1]	94.5%	94.5%	94.6%	94.5%	90.7%	90.4%
Average read depth on target	105x	80x	86x	97x	65x	73x
Number of previously unannotated variants	3,954	3,429	3,922	3,932	3,723	4,022
Novel variants with predicted functional effect[2]	238	189	251	262	541	664
Shared candidate genes (recessive trait)[3]	none	none	none	none	none	none
Genes with putative *de novo* variants[4]	46	-	-	7	?	?
Shared genes with putative *de novo* variants[5]	1	-	-	1	1	1

[1]Referred to Nimblegen SeqCap EZ Library v3.0 (depth ≥ 2).

[2]Filtered on predicted 'missense', 'frameshift', 'stopgain', 'stoploss' and 'splicing' variants

[3]Filtering by requiring the presence of previously unannotated, functionally relevant (*i.e.*, missense, nonsense and splice site changes, and coding indels) and predicted to be disease causing variants in at least two affected subjects and taking into account the presence of the trio.

[4]Filtering by requiring the presence of previously unannotated, functionally relevant (*i.e.*, missense, nonsense and splice site changes, and coding indels) and predicted to be disease causing variants and taking into account the presence of the trio.

[5]Filtering by requiring the presence of previously unannotated, functionally relevant (*i.e.*, missense, nonsense and splice site changes, and coding indels) and predicted to be disease causing variants in all affected subjects and taking into account the presence of the trio.

Supplementary Figure 1. Evolutionary conserved position 446 in *TBL1XR1*.

Homo sapiens	438	LTKHQEPVYSVAFSPDG
Pan troglodytes	438	LTKHQEPVYSVAFSPDG
Rattus norvegicus	438	LTKHQEPVYSVAFSPDG
Canis lupus	438	LTKHQEPVYSVAFSPDG
Bos taurus	438	LTKHQEPVYSVAFSPDG
Monodelphis domestica	439	LTKHQEPVYSVAFSPDG
Gallus gallus	437	LTKHQEPVYSVAFSPDG
Xenopus laevis	446	LTKHQEPVYSVAFSPDG
Malus domestica	484	FDGHRDPVYSVAFSPDG
Zea mays	522	LSGHRQPVYSVAFSPDG
Dictyostelium purpurea	460	LNKHNDPVYTVAFSPNG
Saccharomyces cerevisiae	438	SIVDGVPIFAGRISQDG
